# Eosinophilic esophagitis in an octogenarian: A case report and review of the literature

**DOI:** 10.1097/MD.0000000000005169

**Published:** 2016-10-14

**Authors:** Anca Trifan, Oana Stoica, Catalin-Alexandru Chihaia, Mihai Danciu, Carol Stanciu, Ana-Maria Singeap

**Affiliations:** a “Gr T Popa” University of Medicine and Pharmacy; b “St. Spiridon” Emergency Hospital, Institute of Gastroenterology and Hepatology, Iasi, Romania.

**Keywords:** corticosteroids, dietary therapy, dysphagia, eosinophilic esophagitis, food allergy

## Abstract

**Introduction:**

Eosinophilic esophagitis (EoE) is a chronic, immune/antigen-mediated disease characterized clinically by symptoms related to esophageal dysfunction and histologically by a marked eosinophilic infiltrate in the esophageal mucosa. What was once considered a rare disease has nowadays become one of the most frequent esophageal diseases in the Western countries, occupying a place just next to the gastroesophageal reflux disease. EoE etiology and pathogenesis remain largely unknown, although most studies consider that allergic and genetic factors play the most important role.

**Methods:**

We report the case of EoE in an elderly male (octogenarian), giving a brief review of the current data related to epidemiology, pathogenesis, diagnosis, and treatment of the disease.

**Results:**

Dysphagia to solid foods was the leading symptom, and endoscopic findings included white exudates, longitudinal furrows, and concentric mucosal rings, all suggestive for EoE. Diagnosis relied on histological findings in esophageal mucosal biopsies (>30 eosinophils per high power field).

He was treated with topical steroids for 8 weeks, symptoms improved gradually and the patient remained in remission at the 8-month follow-up.

**Conclusion:**

This case emphasizes that EoE may occur in very old patients and gastroenterologists should have a high index of suspicion of this disorder in any elderly with dysphagia and endoscopic relevant features.

## 1 Introduction

Eosinophilic esophagitis (EoE) was defined by an international expert panel as a chronic, immune/antigen-mediated, esophageal disease characterized clinically by symptoms related to esophageal dysfunction and histologically by a marked esophageal eosinophilic inflammation.^[1]^ Although there were a few case reports of esophageal eosinophilia associated with achalasia or eosinophilic gastroenteritis, published in late 1970s and early 1980s,^[2,3]^ EoE was described only a decade later as a distinct clinicopathological disease.^[4]^ In recent years, EoE has become an increasingly recognized disease discussed at all major international gastroenterology meetings, with an exponential increase in the number of related publications (nearly 1200 PubMed). In addition to a genuine increase, the dramatic growth in the incidence of EoE registered over the last decade throughout the Western world (Europe and North America) is without a doubt influenced by an increased clinical awareness and a large number of esophageal biopsies carried out during endoscopic examinations.^[5]^ EoE is uncommon in elderly patients (aged >65 years), and upon a thorough research, we were able to find only very few case reports in octogenarians in the published literature.^[6]^ EoE is a progressive disease, with a significant impact on the quality of life. Unrecognized and untreated, it may lead to irreversible structural lesions of the esophagus. We hereby present a case of EoE in an elderly male, briefly reviewing the current data on EoE epidemiology, pathogenesis, diagnosis, and treatment.

## 2 Case report

An 89-year-old Caucasian man was referred to our institution in October 2015 with a 1-year history of dysphagia for solid-food, chest pain, and (often) sensation of food impaction which resolved after ingestion of fluids. He did not have pyrosis or acid regurgitation, nor did he have a significant medical history (without any particular history of allergic disease). His symptoms worsened over the past 6 months, but his weight remained stable during this time. Physical examination was unremarkable, electrocardiogram was normal, hematological and biochemical parameters were within normal limits. For his symptoms, he had undergone an upper endoscopy examination performed at another hospital, but he was unable to produce any documentation of this investigation. He remembers, however, only that “the doctor assured him that everything was ok” and prescribed pantoprazole 40 mg/d which he has followed for 3 months with no results to relieve his symptoms.

**Figure 1:**
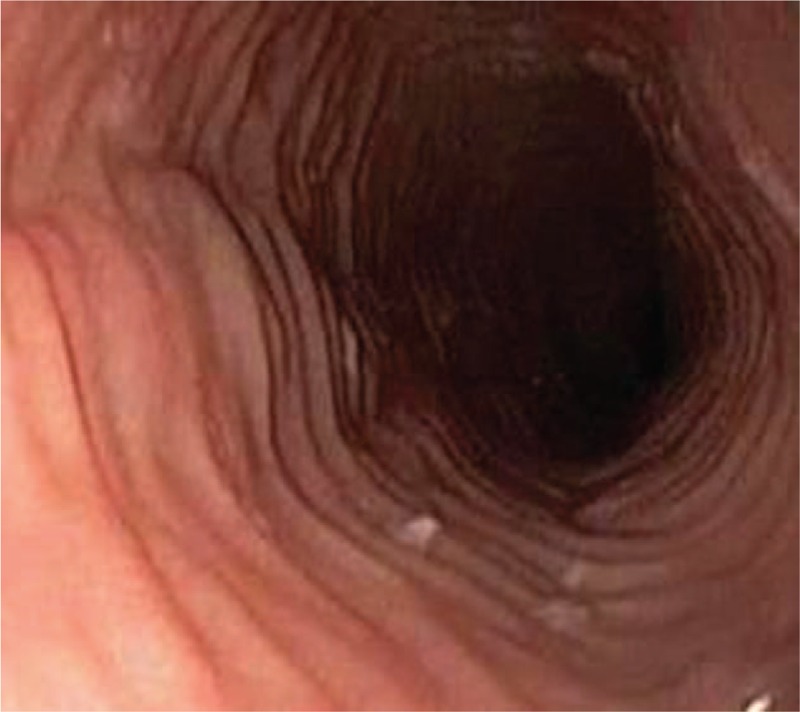
Endoscopic view of the esophagus: prominent fixed esophageal rings (trachea-like aspect).

**Figure 2:**
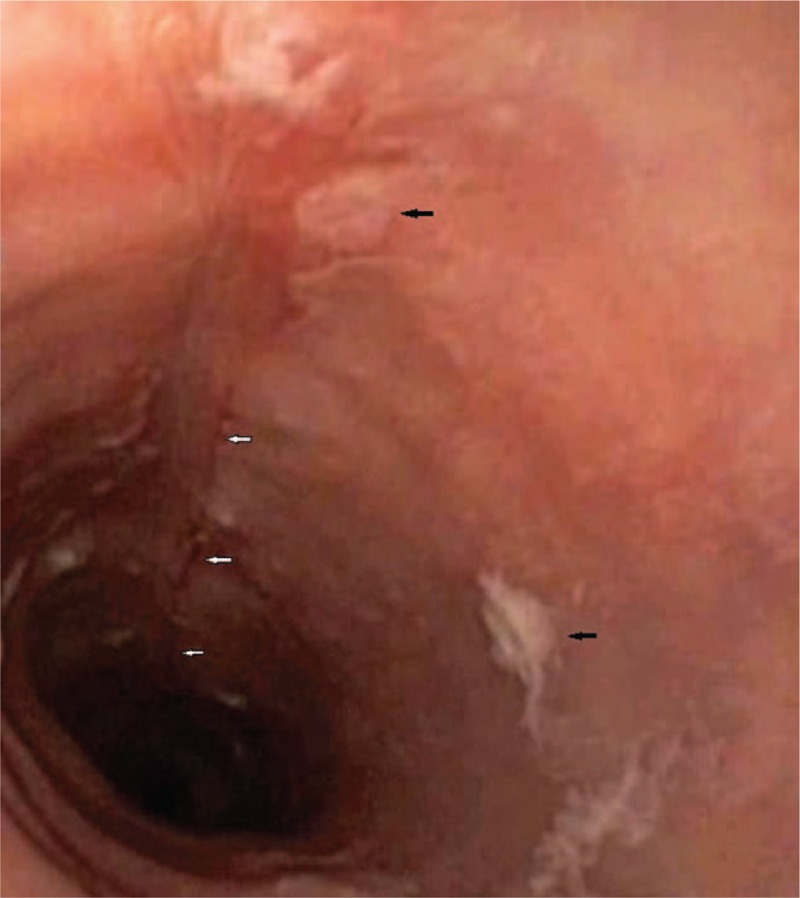
Endoscopic view showing longitudinal linear furrow (white arrows) and white exudates (black arrows).

**Figure 3:**
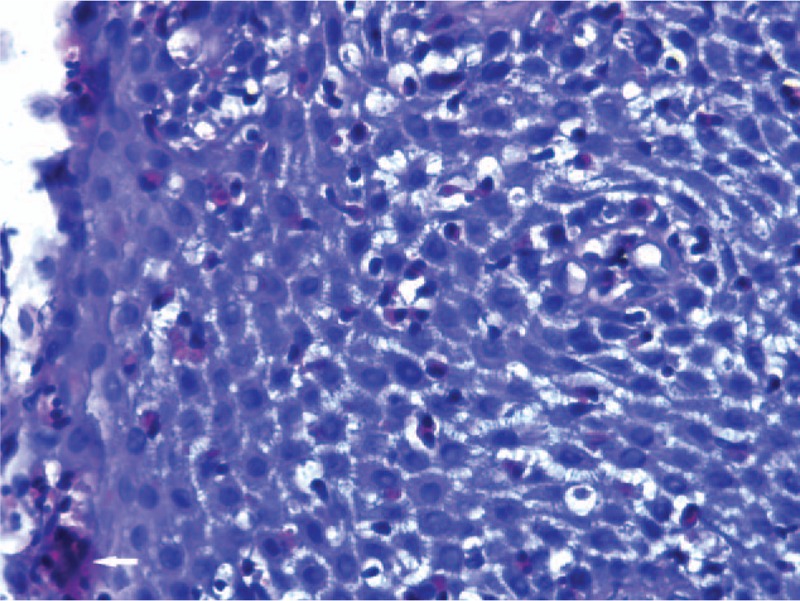
Proximal esophageal biopsy: high power field (HPF) showing a heavy eosinophilic infiltration, >30 eosinophils/HPF (HE, ×400 magnification), and a microabscess formation (clustering of more than 5 eosinophilis) (white arrow).

The upper endoscopy examination was repeated at our institution, showing white exudates, longitudinal furrows, and concentric rings, all suggestive for EoE (Figs. 1 and 2). Biopsy specimens were obtained from proximal and distal esophagus; histologic examination revealed marked infiltration of more than 30 eosinophilis/high power field [HPF] (Fig. 3), confirming the diagnosis of EoE.

Based on clinical symptomatology (and lack of response to the 3-month proton pump inhibitors [PPI] therapy), as well as endoscopic and histologic findings, the patient was diagnosed with EoE. He was treated with fluticasone 250 μg multiple-dose inhaler, 4 puffs swallowed twice a day for 8 weeks. Symptoms improved gradually, and an upper endoscopy performed after 3 months showed marked improvement in endoscopic appearance. The patient remained in remission at the 8-month follow-up.

## 3 Discussion

Although EoE was first described in adults, it has been known best as a children's disease until recently, when several studies and a meta-analysis showed that it is more common in adults.^[5,7–9]^ EoE has been reported at any age, predominantly in the male sex, with a male/female ratio of 3:1.^[10]^ Most frequently, diagnosis is established in the 2nd through 5th decade of life, while cases occurring in very old subjects (octogenarians) have rarely been included in some studies.^[7,8,11,12]^ In our patient, the diagnosis was suggested by clinical symptomatology (dysphagia unresponsive to high-dose PPI therapy) and endoscopic findings (rings, furrows, and white exudates), subsequently confirmed by fulfilling histologic criteria (>15 eosinophils/HPF, bazal—zone hyperplasia, aggregates of eosinophils).^[1,13]^ In addition to his advanced age, another characteristic was his lack of personal or family history of allergic diseases such as asthma, atopic dermatitis, rhinitis, and food allergies which are present in up to 80% of patients with EoE.^[14]^ The patient was treated successfully with topical corticosteroids. In the following, we will summarize the current data on EoE epidemiology, pathogenesis, diagnosis, and treatment.

### 3.1 Epidemiology, pathogenesis

Over the past decade, there has been a dramatic increase in both incidence and prevalence of EoE reported in Europe, North America, Asia (including Japan), and Australia in children as well as adults. Thus, initially known as a rare disease, EoE has nowadays become one of the most frequent esophageal disorders placed next to gastroesophageal reflux disease (GERD). A recent meta-analysis reported pooled incidence and prevalence rates of 3.7/100,000 persons/year and 22.7 cases/100,000 inhabitants, respectively.^[9]^ Using the new international classification of diseases, 9th revision code (ICD-9), Dellon et al^[15]^ found the USA prevalence estimate for EoE of 56.7/100,000 persons. In Canada, 1 study reported a 5-fold increase in the incidence of EoE,^[5]^ while a 19.5-fold rise in the incidence of EoE from 1992 to 2012 was reported in Denmark.^[16]^ Van Rhijn et al^[8]^ estimated the incidence rates of EoE from 1996 to 2010 using a large database in The Netherlands and found a significant increase over the years, being 0.01 in 1996, 0.14 in 2005, and 1.31 per 100,000 persons in 2010, with the highest incidence in 20- to 29-year-old males, and the lowest in the decade 80 to 89 years. This overwhelming growth in the incidence of EoE is most likely influenced by higher clinical awareness and increased number of esophageal biopsies taken during endoscopic examinations^[5]^ rather than being the result of only a genuine increase. Interestingly, the overall incidence and prevalence were higher in adults than in children.^[7–9]^ Thus, considered for several decades as a pediatric disease, EoE has mostly become an adult disease. The etiology of EoE remains largely unknown, although most studies mention that allergic and genetic factors play the most important role.^[17]^ Recent studies consider EoE to be a genetic disease. Thus, EoE genome-wide association studies identified 5 susceptibility loci, among which 2 are specific to EoE.^[18,19]^ Pathogenesis also remains largely unknown. EoE is considered an allergic disease, activated by food allergens and aeroallergens, the allergic mechanism involving both Immunoglobulin E (IgE)-mediated (immediate type) and non-IgE-mediated (delayed type) processes.^[20]^ In the non-IgE-mediated reaction, allergen uptake in the esophagus activates a T helper 2 cell (Th2)-associated immunologic pathway with increased levels of Th2 cytokines interleukin (IL-5) and IL-13, the *eotaxin-3* gene serving as an eosinophilic chemoattractant causing eosinophilic infiltration and inflammation.^[21]^ In the IgE-mediated reaction, mast cells play the important role in the inflammatory process.^[20]^

### 3.2 Diagnosis

The following diagnostic criteria for EoE have been proposed by updated consensus recommendations ^[1]^ and recent guidelines^[13]^: the presence of symptoms related to esophageal dysfunction (dysphagia, food bolus impaction and thoracic pain in adults, and unspecific symptoms in children such as feeding dysfunction, abdominal pain, or vomiting); the presence of minimum 15 eosinophils per HPF/400× magnification in at least 1 esophageal biopsy^[1]^; the persistence of symptoms and/or mucosal esophageal eosinophilia after a 2-month PPI trial^[1,22]^; and secondary causes of esophageal eosinophilia (GERD, eosinophilic gastroenteritis, hypereosinophilic syndrome, Crohn disease, celiac disease, parasitic infection, collagen vascular diseases, and drugs) have been excluded. A distinctive feature of esophageal eosinophilia is that it is not unitary: more than 3 biopsies should be taken from the upper, middle, and lower parts of esophagus.^[23]^

The most important differential diagnosis of EoE is GERD. The clinical history (pyrosis and acid regurgitation), response to PPI, and histology (<10 eosinophils/HPF limited to the distal esophagus) suggest GERD. In contrast to GERD, it was initially assumed that patients with EoE do not response to PPI therapy, and therefore a 2-month PPI trial with a double dose was recommended to distinguish patients with EoE from those with GERD. However, the use of this “diagnostic PPI-trial” has shown that a subset of patients with EoE (approximately 30%) respond both clinically and histologically to treatment with PPI,^[24,25]^ and the term “proton pump inhibitor-responsive esophageal eosinophilia” (PPI-REE) has been proposed and included in recent guidelines.^[1,13]^ Nowadays, PPI-REE is considered a subphenotype of EoE that respond to PPI therapy.^[15,26]^

Endoscopic findings in EoE are multiple, non-specific, and include concentric fixed mucosal rings (“trachea-like aspect”), longitudinal linear furrows, white exudates, fragile, or “*crêpe-paper*” esophageal mucosa appearance, decreased mucosal vascularity or edema, narrowing, and strictures.^[1,13,27]^ There had been a lack of agreement between endoscopists in reporting diagnostic endoscopic features of EoE, until Hirano et al^[28]^ proposed the EoE Endoscopic Reference Score (EREFS) as a method of standardizing description and reporting endoscopic features of EoE, validated recently by Dellon et al^[29]^ as a valuable tool for the diagnosis of EoE. It should be underlined that none of the abovementioned endoscopic features of EoE are part of its diagnosis criteria.^[1,13]^ Moreover, endoscopically normal esophagus is found in up to 30% of patients with EoE.^[22]^

### 3.3 Treatment

#### 3.3.1 Medical therapy

Presently, there are no US Food and Drug Administration and European Medical Agency–approved medical therapy.

*Corticosteroids:* Swallowed topical corticosteroids (fluticasone, budesonide, and ciclosenide) are effective for inducing clinical, endoscopic, and histologic remission^[30]^ and are currently considered as first-line medical therapy for adults with EoE.^[13]^ The use of topical fluticasone (220–880 μg twice daily) or budesonide (1–2 mg once a day or twice a day) for 4 to 6 weeks in randomized, controlled studies resulted in significant improvement in both clinical symptomatology and histology.^[30,31]^ Clinical and histologic remission is followed by maintenance therapy (the duration and optimal dose of which are not yet established). Patients should avoid eating and drinking for at least half an hour after administration of topical corticosteroids. However, almost all patients relapse after cessation of topical steroids. The main side effect of swallowed topical corticosteroids is the infection of the esophagus or oropharyngeal cavity with *Candida albicans,* occurring in 10% to 15% of patients.^[1]^

*Leukotriene inhibitors and immunosuppressants* (purine-analogs) did not prove to have any beneficial effects.^[1,32,33]^

*Biological agents*: A trial performed with anti-IL-5 monoclonal antibody showed a significant reduction of eosinophils in the esophageal tissue and may play an important role in the future therapy of EoE.^[34]^

#### 3.3.2 Elimination and elemental diet therapy

Dietary treatment includes elemental diets, consisting of amino acids–based formulas, which eliminate all potential triggers, and elimination diets, which can be either empirical elimination diets based on avoidance of most common triggers (i.e., milk, egg, soy, wheat, peanuts/tree nuts, and fish/shellfish) or test-directed (targeted) elimination diets, based on allergy test results.

Elemental diets are used to induce rapid disease remission (amino acids formula is not for permanent use).^[35,36]^ The major drawbacks of elemental diets are the very poor taste (children frequently need nasogastric feeding for delivery of amino acids formula).

Empirical elimination diets: the most frequently used is the empiric six food elimination diet (SFED): cow's milk, wheat, egg, soy, peanut/tree nuts, and fish/shellfish are eliminated^[37,38]^ with the advantage that it does not require allergy testing and it allows the consumption of multiple table foods.

Test-directed elimination diets use allergy tests to identify EoE triggers. However, in practice, allergy tests are of very limited value and, therefore, their usefulness remains questionable.^[39]^

#### 3.3.3 Esophageal dilation

Esophageal dilation is indicated in the presence of irreversible fibrostenotic stricture. Many physicians are fearful to dilate the strictures in EoE patients because of early reports^[40,41]^ of severe complications (perforations and mucosal tears). However, recent studies (after 2010) have reported good results and very low rate of complications.^[13,42]^

## 4 Conclusion

Our case report emphasizes that EoE may occur in very old patients, and gastroenterologists should have a high index of suspicion for this esophageal disorder in any elderly adult with clinical symptoms such as dysphagia and food impaction that does not respond to high-dose PPI therapy and in the presence of endoscopic relevant features (furrows, rings, and white exudates). Biopsies should be taken at least from proximal and distal parts of the esophagus to confirm diagnosis.

## Acknowledgments

We thank the patient who agreed to the publication of this case report. A copy of the written consent (in Romanian language) is available for review by the Editor-in-Chief of this journal.
